# Evaluation of a new beads reflux control microcatheter in drug-eluting bead transarterial chemoembolization

**DOI:** 10.1016/j.redii.2024.100048

**Published:** 2024-04-26

**Authors:** Youssef Zaarour, Haytham Derbel, Charles Tran, Laetitia Saccentia, Benjamin Longère, Maxime Blain, Giuliana Amaddeo, Alain Luciani, Hicham Kobeiter, Vania Tacher

**Affiliations:** a Department of Radiology, CHU Henri-Mondor, Assistance publique – hôpitaux de Paris (AP-HP), 1, rue Gustave-Eiffel, 94010 Créteil, France; b Université Paris-Est Créteil (Upec), 94010 Créteil, France; c Unité Inserm U955, équipe n°18, IMRB, 94010 Créteil, France; d Department of Cardiovascular Radiology, Institut Cœur-Poumon, CHU de Lille, 59037 Lille, France; e Department of Hepatology, CHU Henri-Mondor, Assistance publique – hôpitaux de Paris (AP-HP), 94010 Créteil, France

**Keywords:** Reflux control, Microcatheter, DEB-TACE, Hepatocellular carcinoma, Beads

## Abstract

**Rationale and objectives:**

A new microcatheter was recently developed claiming to reduce beads reflux in drug-eluting bead transarterial chemoembolization (DEB-TACE). The aim of this study was to compare the reflux control microcatheter ability versus a standard microcatheter for TACE treatment in patients with hepatocellular carcinoma.

**Material and methods:**

Patients were prospectively included between November 2017 and February 2022. They received a DEB-TACE treatment with charged radiopaque beads using standard microcatheters or the SeQure reflux control microcatheter (Guerbet, France) and were assigned respectively to a control and a test group. Beads distribution mismatch was evaluated between the targeted territory on treatment planning CBCT and beads’ spontaneous opacities on the post-intervention CBCT and the 1-month CT scanner.

**Results:**

Twenty-three patients (21 men, median age 64 years [12.5 years]) with 37 hepatocellular carcinoma nodules were treated. The control group consisted of 13 patients – 19 nodules, while the test group included ten patients - 18 nodules. Non target embolization (NTE) was found in 20 % (2/10) of patients in the test group and 85 % (11/13) in the control group. NTE involved only an adjacent segment in the test group while it affected the adjacent biliary sector or even the contralateral liver lobe in the control group. No complication linked to NTE was found in the test group, while it led to one case of ischemic cholangitis and another case of biloma in the control group.

**Conclusion:**

The reflux control microcatheter may be efficient in reducing NTE and thus eventual adverse events in comparison to standard of care end-hole microcatheters.

## Graphical abstract

**Figure:**
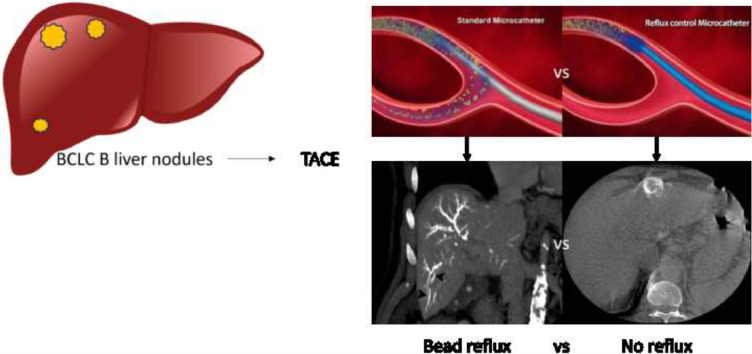

## 1 Introduction

Liver cancer is the third most frequent cause of cancer-related death around the globe. Hepatocellular carcinoma (HCC) is by far the most common type of primary liver cancers (75 to 85 %) [1], and the most common risk factor is liver cirrhosis.

Transarterial chemoembolization (TACE) is the recommended treatment for intermediate stage HCC [2] when curative options are not available. In conventional TACE (cTACE), the chemotherapeutic agent, most frequently doxorubicin, is injected in an emulsion with Lipiodol® (Guerbet, Villepinte, France) into the branch of the hepatic artery feeding the tumor [3], followed by the injection of an embolic material [4]. In a more recent TACE technique, called drug-eluting bead TACE (DEB-TACE) [5], calibrated beads are loaded with chemotherapeutics beforehand, so as to provide a more consistent embolization and a more controlled and sustained delivery of the drug.

Antireflux or flow directed microcatheters have been developed and marketed since a few years, aiming to “avoid” non target embolization and increase drug and bead delivery to the target lesion [6,7].Those devices rely on mechanically occluding the feeding artery through most commonly a distal tip balloon-occlusion microcatheter, thus preventing reflux and increasing beads penetration into the target lesion due to reduced downstream blood pressure following the obstruction. Their use is however debated due to cases of iatrogenic arterial wall injury [8,9] and potential opening of interlobar and intersegmental vascular shunts [7,10].A new reflux control microcatheter (SeQure Guerbet company, France) has recently been developed. It is different from traditional antireflux microcatheters with a view to preventing beads reflux by creating a fluid barrier of turbulent flow at the distal end of the microcatheter through microscopic side holes [11], without any occluding device or obstruction of the natural arterial flow. It has been tested in vitro and in vivo using regular, radiolucent beads and obtained FDA approval in January 2018 and CE mark approval in April 2019 [12].

However, most beads are radiolucent and have to be mixed with contrast media allowing only an indirect visualization of the embolic material, making postintervention target evaluation and beads distribution rather difficult to distinguish from contrast trapping [13]. In the past years, radiopaque beads have been developed to provide intraprocedural feedback on treated locations, to help avoid nontarget embolization, and to better evaluate tumor embolization [14,15].

The main objective of this study was to assess the effectiveness of the SeQure microcatheter by using it with radiopaque beads to evaluate its reflux control properties compared with a standard-of-care catheter for nontarget embolization during DEB-TACE procedures. Secondary objectives were the evaluation of tumor coverage by the embolic agents during and following the procedure, as well as tumor response and adverse events.

## 2 Methods

### 2.1 Patients

We prospectively included all patients with focal or multifocal HCC, either primary or recurrent, who were candidates for a DEB-TACE treatment between November 2017 and February 2022 in our department. The institutional review board approval was obtained (IRB approval number: CRM-2206–278). All the procedures were performed in compliance of the ethical standards adopted by our institution's research and ethics committee, the 1964 Helsinki declaration and its subsequent amendments. The study was assessed using STROBE (STrengthening the Reporting of OBservationalv studies in Epidemiology) guidelines [16]. The patients were selected for TACE treatment after their consent and following multidisciplinary consensus, in accordance with current established guidelines for the management of HCC as mentioned below. The multidisciplinary tumor board included oncology, hepatology, visceral and digestive surgery, radiation oncology, nuclear medicine, pathology, and diagnostic radiology specialists. TACE treatment was then planned during an interventional radiology staff meeting. Criteria for receiving TACE treatment included HCC, absence of extrahepatic spread, absence of complete portal occlusion, Eastern Cooperative Oncology Group (ECOG) performance status ≤ 1, and Child-Pugh class A or B7, all in accordance to the Barcelona Clinic Liver Cancer (BCLC) guidelines [17], intermediate stage of the European Association for the Study of Liver (EASL) classification [2] and the European Society of Medical Oncology (ESMO) guidelines [18]. Patients were excluded if they had undergone prior TACE treatment with radiopaque material of the same nodule or segment.

The choice of DEB-TACE over cTACE was made during the interventional radiology staff meeting for the sole purpose of this study as the main chemoembolization intervention at our institution is cTACE. Patients were eligible for DEB-TACE if superselective catheterization (subsegmental or beyond) of the feeding artery was deemed possible as the radiopaque beads (DC Bead LUMI™, Boston Scientific, Massachusetts USA) used in this technique measure 70 to 150 μm and thus convey a higher risk of biliary complications [19,20]. All their medical records were reviewed and analyzed.

Three hundred and eighty-two patients received a TACE treatment at our institution during the inclusion period.

**Fig. 1:**
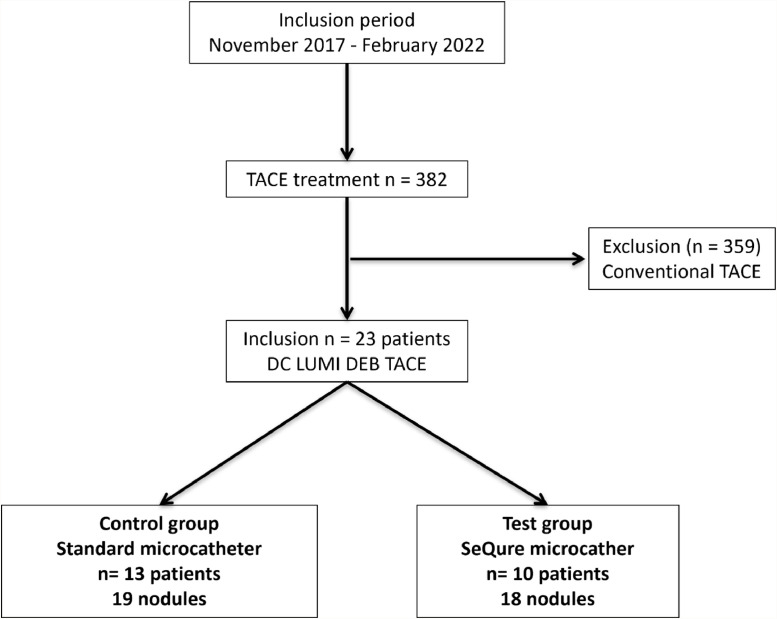
Comparison of the reflux control microcatheter ability versus a standard microcatheter for transarterial chemoembolization (TACE) treatment in patients with hepatocellular carcinoma: Flow diagram illustrating the inclusion process of the study. DEB: drug-eluting bead.

Among them, only 23 patients had a DEB-TACE and were included in the study (Fig. 1), 21 (21/23, 96 %) of whom were male. The median age was 64 years old (25th percentile Q1: 62 years, 75th percentile Q3: 74 years). All but one had comorbidities including hypertension (61 %); type 2 diabetes (44 %); past or current tobacco smoking (59 %); cardiopathy (22 %); and a history of another neoplasm (26 %).

Twenty-one patients (96 %) had underlying liver cirrhosis, most frequently multifactorial, and 16 of these patients (74 %) had a history of alcohol-related liver disease.

The HCC was unifocal in ten patients (44 %) and de novo in 17 (74 %). The Child-Pugh score was A in 14 patients (61 %), and B7 in nine (39 %). Treatment by TACE was recommended for 12 patients (52 %) who were classified as BCLC B, and for ten patients (44 %) with BCLC A and one patient (4 %) with BCLC 0 as a bridge to liver transplantation.

Five patients (21 %) had undergone prior cTACE, six patients (25 %) a prior surgical resection, four (17 %) had percutaneous thermal ablation, all in a different liver segment from the nodule treated. One patient (4 %) had received previous systemic chemotherapy.

The patients were consecutively and alternatively assigned into two groups based on the microcatheter used: the test group for the SeQure microcatheter, and the control group for standard-of-care, end-hole, microcatheter.

The test group was composed of ten patients with 18 nodules, and the control group of 13 patients with 19 nodules (Fig. 1). Three additional patients were added in the control group to reach an equivalent number of treated nodules in each group.

Patient demographics and characteristics are detailed in Table 1.

**Table 1:** Patient characteristics.

	**Total** (***n*****= 23)**	**Control group (*****n*****= 13)**	**Test group (*****n*****= 10)**	*p*
*Sex* n *(ratio,%)*				0.21
Male	21 (21/23; 91 %)	11 (11/13; 85 %)	10 (10/10; 100 %)	
Female	2 (2/23, 9 %)	2 (2/13; 15 %)	0	
*Age years*	(64; 62, 74)	63; 62, 67	70; 61, 75	0.46
*Body* m*ass* i*ndex (mean ± SD), kg/m²*	(28; 24, 33)	26; 24, 31	30; 25, 34	0.29
*ECOG* n *(ratio,%)*				0.37
0	14 (14/23; 61 %)	9 (9/13, 69 %)	5 (5/10, 50 %)	
1	9 (9/23; 39 %)	4 (4/13, 31 %)	5, (5/10, 50 %)	
≥ 2	0 (0 %)			
*Comorbidities* n *(ratio,%)*				
Hypertension	14 (14/23; 61 %)	9 (9/13, 69 %)	5 (5/10, 50 %)	0.37
Type 2 diabetes	10 (10/23; 44 %)	4 (4/13, 31 %)	6 (6/10, 60 %)	0.18
Tobacco smoking	13 (13/23; 59 %)	8 (8/13, 62 %)	5 (5/10, 50 %)	0.6
Cardiopathy	5 (5/23; 22 %)	2 (2/13; 15 %)	3 (3/10, 30 %)	0.42
Other cancers	6 (6/23; 26 %)	3 (3/13, 23 %)	3 (3/10, 30 %)	0.72
*Liver cirrhosis,* n *(ratio,%)*	22 (22/23; 96 %)	12 (12/13, 92 %)	10 (10/10, 100 %)	0.39
*Cause of liver cirrhosis* n *(ratio,%)*				
Alcohol-related liver disease	17 (17/23; 74 %)	8 (8/13, 62 %)	9 (9/10, 90 %)	0.13
Non-alcoholic steatohepatitis	10 (10/23; 44 %)	5 (5/13, 38 %)	5 (5/10, 50 %)	0.6
Viral hepatitis	6 (6/23; 26 %)	5 (5/13, 38 %)	1 (1/10, 10 %)	0.13
Primary biliary cholangitis	1 (1/23; 4 %)	1 (1/13, 8 %)	0	0.39
*Characteristics of HCC* n *(ratio,%)*				
Unilocular	10 (10/23; 44 %)	8 (8/13, 62 %)	2 (2/10, 20 %)	0.05
Multifocal	13 (13/23; 57 %)	5 (5/13, 38 %)	8 (8/10, 80 %)	0.05
De novo	17 (17/23; 74 %)	9 (9/13, 69 %)	8 (8/10, 80 %)	0.6
Recurrent	6 (6/23; 26 %)	4 (4/13, 31 %)	2 (2/10, 20 %)	0.6
*BCLC* n *(ratio,%)*				
0	1 (1/23; 4 %)	1 (1/13, 8 %)	0	0.39
A	10 (10/23; 44 %)	6 (6/13, 46 %)	4 (4/10, 40 %)	0.78
B	12 (12/23; 52 %)	6 (6/13, 46 %)	6 (6/10, 60 %)	0.53
C	0 (0/23; 0 %)	0	0	N/A
*Child-Pugh* n *(ratio,%)*				
A	14 (14/23, 61 %)	9 (9/13, 69 %)	5 (5/10, 50 %)	0.37
B	9 (9/23; 39 %)	4 (4/13, 31 %)	5 (5/10, 50 %)	0.37
C	0 (0 %)	0	0	N/A
*MELD*	(11; 9, 15)	9; 7, 11	14; 11, 16	0.03
*Prior treatment* n *(ratio,%)*				
TACE	5 (5/23; 21 %)	3 (3/13, 23 %)	2 (2/10, 20 %)	0.78
Surgical resection	6 (6/23; 25 %)	4 (4/13, 31 %)	2 (2/10, 20 %)	0.5
Thermal ablation	4 (4/23; 17 %)	1 (1/13, 8 %)	3 (3/10, 30 %)	0.22
Chemotherapy	1 (1/23; 4 %)	1 (1/13, 8 %)	0	0.37

Qualitative variables are expressed as raw numbers; numbers in parentheses are proportions followed by percentages. Quantitative variables are expressed as medians, followed by first quartiles and third quartiles Q1: first quartile, Q3: third quartile. BCLC: Barcelona Clinic Liver Cancer; ECOG: Eastern Cooperative Oncology Group; HCC: hepatocellular carcinoma; MELD: Model for End-Stage Liver Disease; TACE: transarterial chemoembolization.

### 2.2 Chemoembolization procedure

All the procedures were performed in one of two angiography suites (Azurion® or Allura® Xper FD20 Clarity, Philips Healthcare) equipped with C-arm CBCT (XperCT®, Philips Healthcare) and a dedicated workstation (Release® 8.0, Philips Healthcare) using automatic vessel detection software (Emboguide®, Philips Healthcare).

All DEB-TACE procedures were performed under local anesthesia using 1 % lidocaine. First, the hepatopetal portal flow was checked by indirect portal angiography through the superior mesenteric artery and opacification. After selection of the hepatic artery, digital substraction angiograms and three-dimensional arteriography using cone-beam computational tomography (CBCT) were then acquired to guide catheter navigation.

Dual phase-CBCT (DP-CBCT) was acquired following selection of the hepatic artery initially to create the 3D roadmap and identify the feeding artery then after super-selective selection to confirm the microcatheter position. The acquisition is realized 5 s after injecting 20 ml of contrast media at a 2 ml/s rate into the hepatic artery or 5 ml of contrast mediat at a 0.5 ml/s rate when super-selective, through two, 5 s each, C-arm rotations separated by an interval of 5 s [21]. Advanced image reconstruction allowed CT-like acquisitions and 3D modeling, creating a 3D roadmap overlaid on the 2D fluoroscopy using Emboguide®.

DEB-TACE was performed using 70–150 µm radio-opaque beads preloaded with 75 mg of doxorubicin hydrochloride [22] and suspended during the intervention in Visipaque™ 320 (GE Healthcare, Chicago, Illinois USA) with a dilution of 2 mL of preloaded beads for 30 mL of contrast. Microcatheters used for beads delivery were the standard-of-care end-hole microcatheters 2.7 Fr and 2.4Fr PROGREAT® (Terumo, Tokyo, Japan), 2.4 Fr Direxion™ (Boston Scientific, Marlborough, Massachusetts USA), and the reflux control microcatheter 2.7Fr and 2.4Fr SeQure.

The SeQure microcatheter has side slits measuring less than 70 µm between two radiopaque markers placed at 11 mm from each other on its distal tip allowing lateral outflow of the contrast media and not of the beads thus creating a fluid reflux barrier between the radiopaque markers [23]. This microcatheter, and its reflux control effect, is thus compatible with DC Bead LUMI.

The chemoembolic emulsion was slowly injected at the treatment target point at a rate of approximately 1 mL/min [24]. The treatment endpoint was reached when contrast media and embolic agent stasis was observed after three heartbeats [25].

Beads distribution in the liver was evaluated immediately at the end of the procedure with a non-enhanced CBCT acquisition.

All patients were discharged 24 h after the procedure.

### 2.3 Outcome measures

Beads reflux was assessed using intraoperative non-enhanced CBCT at the end of the procedure (defined as H0) and 1-month non-enhanced CT (NECT) (defined as M1) acquisition. It was defined as the presence of radiopaque material in nontarget segmental branches of the hepatic arteries. The target and nontarget segments were noted on the DP-CBCT, H0 and M1 and the reflux mismatch was evaluated by two radiologists with 3- and 5-years’ experience in hepatic imaging, respectively. Mismatch was considered when vessel-like linear opacities were found at H0 or M1 in comparison to the DP-CBCT, corresponding to nontarget embolization of beads.

**Fig. 2:**
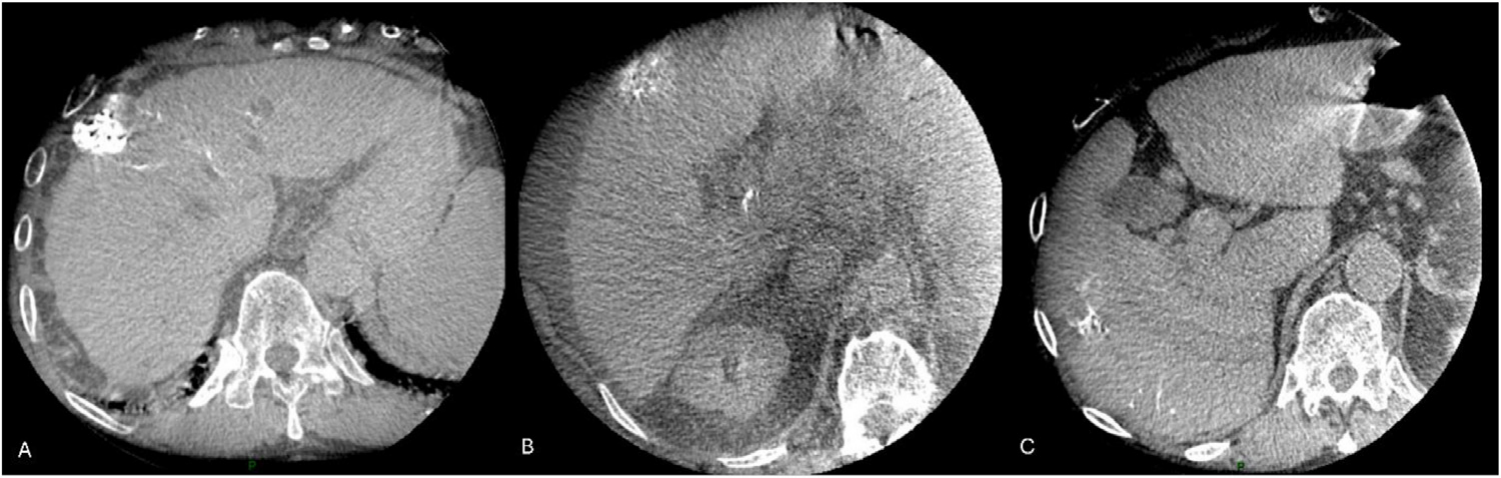
Examples of intratumoral bead distribution according to Aliberti classification [26] with a grade 1 (75 – 100 %) distribution (A), a grade 2 (25 – 75 %) distribution (B) and a grade 3 (<25 %) distribution in two nodules of segments V and VI (C).

Distribution of radiopaque beads in each individual nodule was classified using Aliberti et al.’s initial description [26] in grades from 1 to 3 based upon percentages of beads distribution in the volume of the target nodule (G1 = 75–100 %, G2 = 25–75 %, G3 = 0–25 %), as shown in Fig. 2. Intranodular distribution mismatch was defined as a grading difference between intraoperative CBCT and M1 NECT.

Perprocedural data as dose area product (DAP) in mGy.cm^2^, and fluoroscopy time in minutes were also recorded.

Postprocedural complications were evaluated either clinically as the presence of postembolization syndrome (primarily consisting of postoperative pain, fever, nausea and/or vomiting), or radiologically as the presence of focal liver necrosis and bilomas or signs of ischemic cholangitis (dilated or irregular biliary branches) on M1 multiphase CT and MRI to detect a potential correlation with beads reflux and its location. The adverse event severity scale of the Society of Interventional Radiology (SIR) [27] was used to assess the potential repercussion of these complications on patient management.

Radiological analysis and data collection of both the DP-CBCT and H0 CBCT were made on the Phillips Workstation of the angiography suite, while the M1 CT scan was analyzed on the local PACS (Carestream, Rochester, USA). Biological and clinical information of each patient were collected from the local digital archives.

Tumor response was evaluated on M1 multiphase CT and MRI using modified Response Evaluation Criteria in Solid Tumors (mRECIST) [28] and Liver Imaging-Reporting and Data System (LI-RADS) [29] treatment response algorithms.

### 2.4 Statistical analysis

The Shapiro test was performed to assess the distribution of variables. Quantitative variables that were not normally distributed were expressed as median and quartiles (25th and 75th percentiles) while normally distributed variables were expressed as means and standard deviation. Descriptive data consisting of continuous and categorical variables were described as counts, ratios, and percentages respectively.

Fisher's exact test for univariate analysis of the impact of patient characteristics for beads reflux was performed using SPSS version 27 for Windows (IBM, USA).

P values less than 0.05 were considered significant.

## 3 Results

There was no significant demographic or clinical data difference found between the two groups as shown in Table 1 besides the Model for End-Stage-Liver Disease (MELD) score (*p* = 0.03) and the unifocal/multifocal disease (*p* = 0.05).

Twenty-three patients were treated in 24 interventions targeting 37 nodules. The mean number of HCC nodules was 1.8 nodules (range 1 – 3) in the test group and 1.5 in the control group (range 1 – 3). The mean size of the target lesions was 23 mm in the test group (range 12 – 37 mm) versus 26 mm in the control group (range 9 – 34 mm).

H0 CBT data were missing for one patient in the control group with two nodules. For this patient the 1-month (M1) beads distribution could be analyzed by CT scan but it was not possible to evaluate if distribution had changed between H0 and M1. No patient drop-out or loss to follow-up was noted.

Microcatheter occlusion requiring the use of a new microcatheter occurred during four interventions (36 %, 4 out of 11 interventions) in the test group, and none in the control group. This was prevented for the remaining interventions of the test group by continuous flushing of the microcatheter with contrast media when the emulsion was not being injected.

The mean duration of an intervention was 87 min with an average fluoroscopy time of 26 min. The median dose area product was 175,000 mGy.cm^2^ (134,885; 220,772).

Detailed results of the intervention and the control at M1 are found below and in Table 2.

**Table 2:** Analysis of drug-eluting bead transarterial chemoembolization procedures and subsequent clinical and radiological assessments.

	Control group	Test Group	*p*
*Perprocedural parameters* • Duration of procedure (mean ± SD), minutes 87 ± 26 [45; 140] • Dose-area product (median; Q1, Q3), mGy.cm² (175,000; 134,885, 220,772)			
	76 ± 18 [45; 100]	97 ± 29 [60; 140]	0.05
	(151,822; 113,007, 208,927)	(176,536; 149,153, 286,889)	0.14
*Beads reflux* • *N* = 23 patients • H0 CBCT • M1 CT scanner			
	13	10	0.07
	7 (58 %, 7/12, one missing data)	2 (20 %, 2/10)	<0.01
	11 (85 %, 11/13)	2 (20 %, 2/10)	
*Distribution of beads in nodules* • *N* = 37 nodules • On H0 CBCT ○ G1, *n* = 14 (40 %) ○ G2, *n* = 11 (31 %) ○ G3, *n* = 10 (29 %) • On M1 CT: ○ G1, *n* = 8 (22 %) ○ G2, *n* = 7 (19 %) ○ G3, *n* = 22 (60 %) • Mismatch between H0 and M1, *n* = 13 (37 %)			
	19 nodules missing data in 2 nodules	18 nodules	0.8
	6 (35 %)	8 (44 %)	0.67
	6 (35 %)	5 (28 %)	0.64
	5 (29 %)	5 (28 %)	
	3 (16 %)	5 (28 %)	
	4 (21 %)	3 (17 %)	
	12 (63 %)	10 (55 %)	
	6 (35 %)	7 (39 %)	
*Postprocedural complications* • Yes, *n* = 6 patients (26 %) • Postembolization syndrome, *n* = 1 (4 %) • Biloma, *n* = 3 (13 %) • Ischemic cholangitis, *n* = 2 (9 %) • Death, *n* = 0			
	3 patients	3 patients	
	1	0	
	2	1	
	0	2	
	0	0	
*LI-RADS evaluation of nodules (*n *=37) at M1* • Nonviable, *n* = 17 (46 %) • Viable, *n* = 18 (49 %) • Equivocal, *n* = 2 (6 %)	19 nodules	18 nodules	0.32
	9 (47 %)	7 (39 %)	
	10 (53 %)	9 (50 %)	
	0	2 (11 %)	
*mRECIST evaluation of nodules (*n *=37) at M1* • Complete response, *n* = 17 (46 %) • Partial response, *n* = 13 (35 %) • Stable disease, *n* = 7 (19 %) • Progressive disease, *n* = 0	19 nodules	18 nodules	0.37
	9 (47 %)	8 (44 %)	
	8 (42 %)	5 (28 %)	
	2 (11 %)	5 (28 %)	
	0	0	

Qualitative variables are expressed as raw numbers; numbers in parentheses are proportions followed by percentages. Quantitative variables are expressed as mean ± standard deviation, minimum and maximum values if normally distributed and medians, followed by first quartiles and third quartiles Q1: first quartile, Q3: third quartile if the variable aren't normally distributed. -RADS: Liver Imaging-Reporting and Data System; mRECIST: modified Response Evaluation Criteria in Solid Tumors.

### 3.1 Beads distribution

**Fig. 3:**
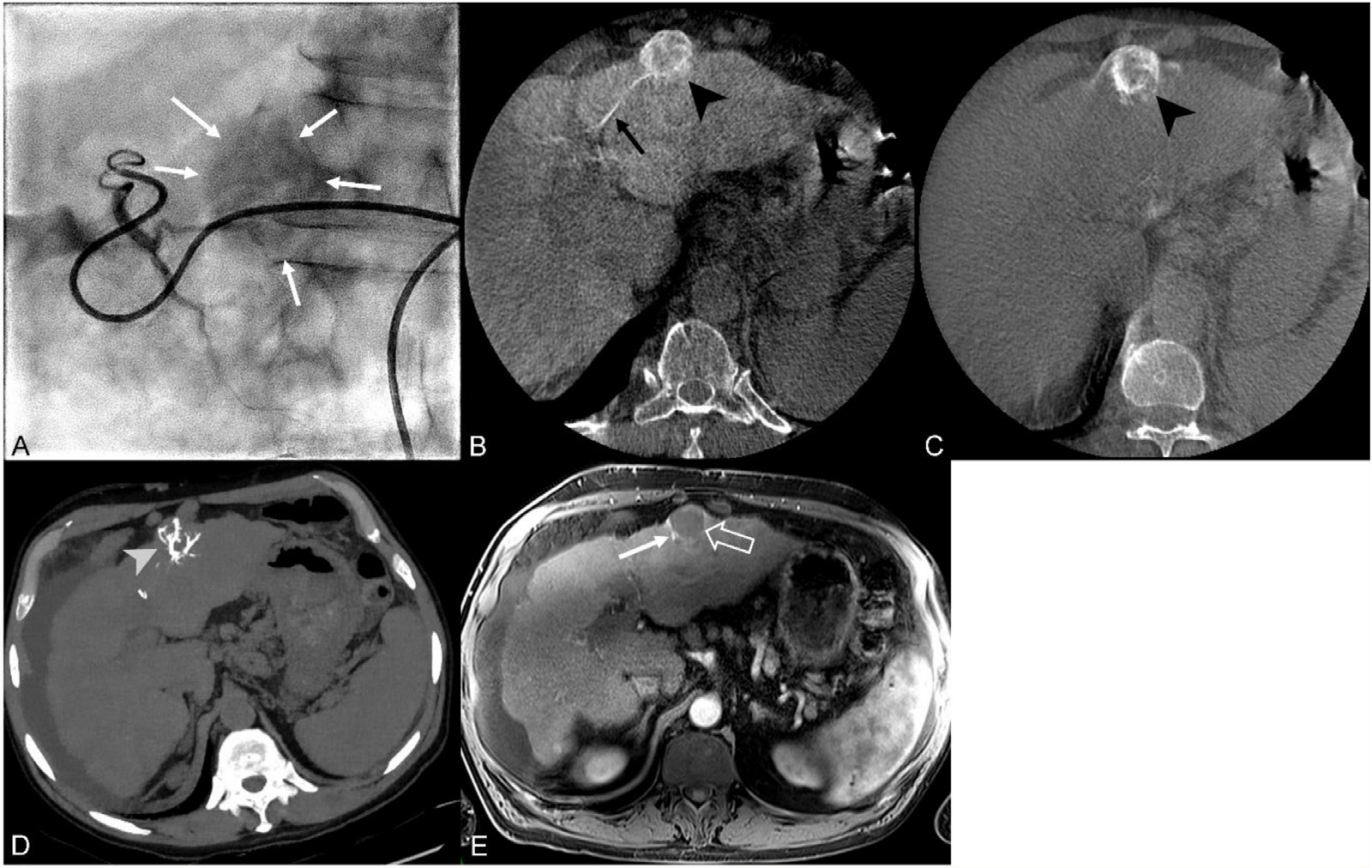
Test gro**up case: a 56**-year-old male with two hepatocellular carcinoma (HCC) nodules of the hepatic segments II and III. Manual hyperselective opacification of segment III (A) using a SeQure microcatheter shows a nodular enhancement (white full arrows). The arterial phase of the contrast enhanced cone beam CT (CBCT) B) shows the feeding artery (black full arrow) and the enhanced subcapsular nodule (black arrowhead). The result of the following drug eluting beads transarterial chemoembolization (DEB-TACE) is a G1 fixation of the nodule at H0 CBCT (C – black arrowhead) with persistent fixation at the M1 non enhanced CT (D – black arrowhead) displayed in maximal intensity projection (MIP). Devascularization of the nodule is shown at the M1 MRI (E – white hollow arrow) with a thin enhancing rim (white full arrow) that disappeared in following controls.

**Fig. 4:**
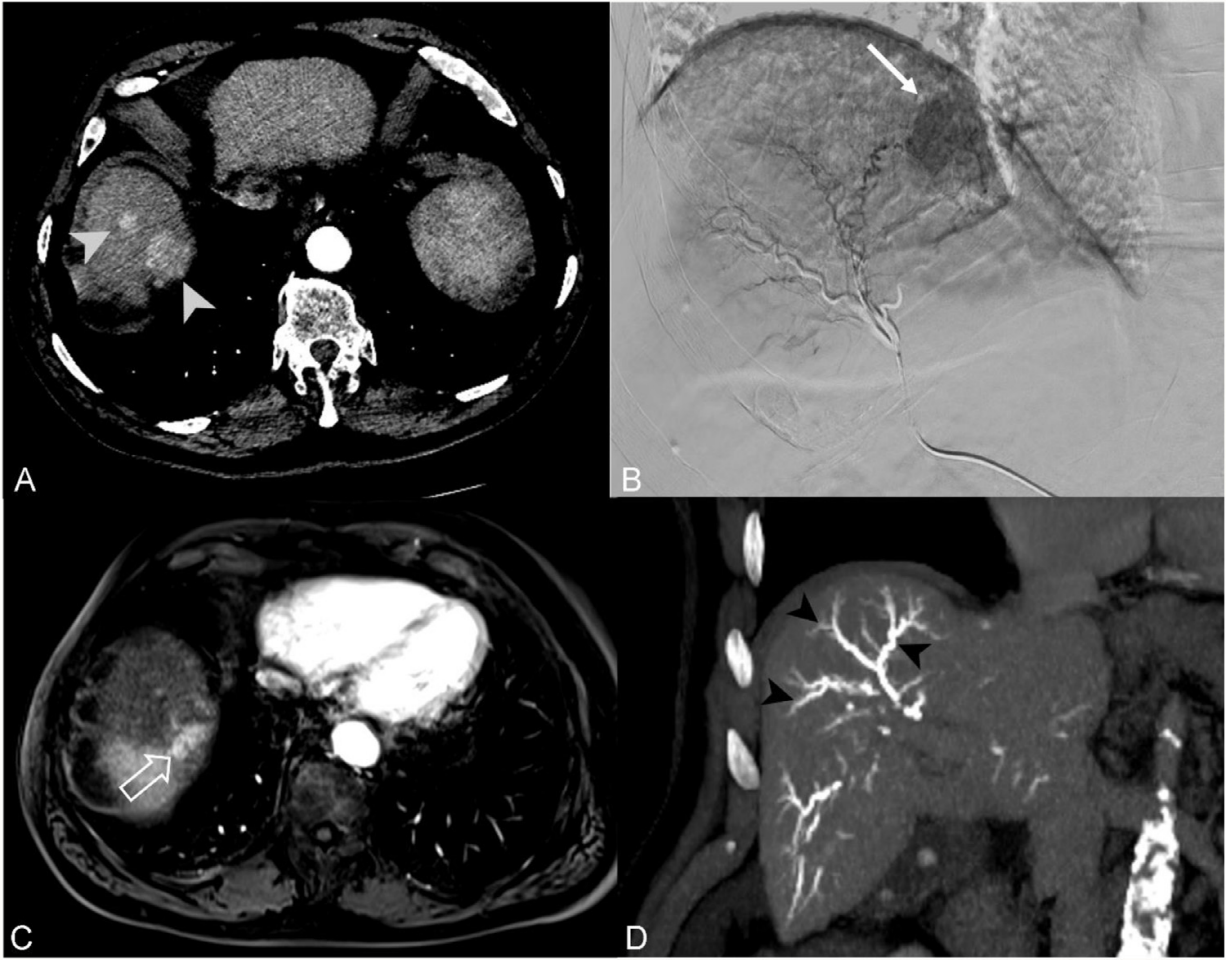
Control group case 1: a 78-year-old male patient presents with two hypervascular HCC nodules of the hepatic dome (A – white arrowheads). Selective angiography of the right hepatic lobe using a standard microcatheter (B) shows the nodular hypervascular nodule (white full arrow) and its feeding artery from hepatic segment VIII before DEB-TACE using DC Bead LUMI. A viable enhancing lesion is found at the M1 control MRI (C – white hollow arrow). Black arrowheads (D) show the beads vascular cast in the targeted hepatic segment VIII while white arrowheads show their reflux in segment V at the non-enhanced CT acquisition after 1 month. DEB-TACE: drug-eluting bead transarterial chemoembolization; HCC: hepatocellular carcinoma.

Overall, beads reflux was observed in 39 % of the patients (9/22) at H0, and in 57 % of patients (13/23) at M1, most frequently in the complementary segment of the same hepatic sector. It occurred in 20 % of the patients (2/10) of the test group (Fig. 3) at H0 with beads found in one adjacent segment compared with DP-CBCT, without changes at M1. In the control group (Fig. 4), 58 % of patients (7/12) showed evidence of beads reflux at H0 and 85 % patients (11/13) at M1, with nontarget embolization observed even in the contralateral liver lobe in 15 % of the patients (2/13). Beads distribution and reflux increased between H0 and M1 in four patients (31 %) in the control group.

Univariate analysis of patient characteristics showed no significant association with beads reflux.

In the 37 nodules treated in all 23 patients (Table 2), H0 and M1 acquisitions indicated that beads distribution was optimal (G1) in 40 % (14/35, two patients with missing data) and 22 % (8/37) nodules, respectively. G2 distribution was observed in 31 % (11/35) and 19 % (7/37) nodules respectively. G3 beads distribution was found respectively in 29 % (10/35), and 60 % (22/37), respectively. No significant difference in beads distribution was found between the control and test groups.

Distribution mismatch between the two evaluations was observed in 37 % of the nodules (13/35), constantly downgraded at M1 and most frequently to G3 (77 %, 10/13).

### 3.2 Adverse events

Clinical monitoring after the procedure reported only one case of immediate postembolization syndrome amongst the patients (4 %).

**Fig. 5:**
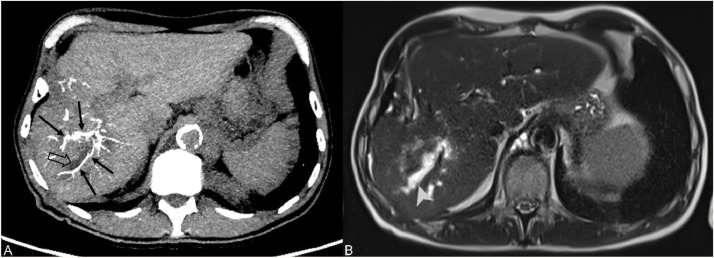
Control group case 2: a 62-year-old male patient from the control group treated with DEB-TACE for an HCC nodule of hepatic segment VII. Non-enhanced CT at M1 showing the spontaneously radiopaque vascular cast of the DC Bead LUMI (A – black full arrows) at the junction of the anterior and posterior liver sectors with a dilated adjacent biliary duct (black empty arrow). Axial T2 Haste sequence (Siemens Healthineers, Germany) at the same level of the liver better show the dilated biliary ducts (B – white arrowhead) in keeping with an ischemic cholangitis following non-target embolization. DEB-TACE: drug-eluting bead transarterial chemoembolization; HCC: hepatocellular carcinoma.

On the radiological follow-up at M1, two cases of biloma (20 %, 2/10) were found in the treated segment in the test group. These remained asymptomatic and were incidentally found without any effect on the treatment plan or the Adverse Effect Severity Scale. However, three patients experienced complications in the control group (23 %, 3/13): two cases of ischemic cholangitis and one biloma. Only one of the cases, a case of ischemic cholangitis, occurred in the treated territory while the others were found at the site of reflux and nontarget embolization (Fig. 5), without any direct or indirect repercussions on the patient or the treatment plan.

### 3.3 Tumor response assessment

Using the mRECIST criteria for index lesions at M1, 17 nodules (46 %) had complete response, 13 (35 %) had partial response, and seven (19 %) had stable disease. Progressive disease was noted in one patient who developed another focus of HCC.

Evaluation using the LI-RADS treatment response algorithm (LR-TR) demonstrated nonviable tumors in 17 of the 37 HCC nodules (46 %), viable tumors in 18 (49 %), and equivocal tumors in two (6 %).

No significant difference was found between the two groups as shown in Table 2.

Integrating beads distribution data in all 17 nodules that were found to be LR-TR nonviable, Aliberti's classification was respectively at H0 and M1: eight (47 %) and six (35.5 %) were rated grade 1, five (29 %) and five (29 %) were rated grade 2, and four (24 %) and six (35.5 %) were rated grade 3.

Among the 18 viable nodules, grading at H0 (missing data for two nodules thus *n* = 16) and M1 (*n* = 18) was respectively: 5/16 (31 %) and 2/18 (11 %) rated as grade 1, 6/16 (38 %) and 1/18 (6 %) as grade 2, and 5/16 (31 %) and 15/18(83 %) as grade 3.

Among the grade 1 nodules, five out of 14 nodules (36 %) at H0 and two out of eight nodules (25 %) at M1 remained viable.

Among a total of 22 nodules rated as grade 3 at M1, six nodules were nonviable (27 %), while one (5 %) was equivocal, and 15 (68 %) remained viable.

A Fisher exact test, evaluating the association between LI-RADS classification and Aliberti's classification, shows a two-sided p-value of 0.015 (< 0.05).

Of the 13 HCC nodules that presented a distribution mismatch between H0 CBCT and M1 NECT evaluation, four (31 %) showed a complete response to treatment, graded LR-TR nonviable.

## 4 Discussion

Beads reflux in DEB TACE is a potential source of complications and is difficult to prevent and to assess. A new anti-beads reflux microcatheter was recently developed. It was used in our study combined with radiopaque beads to assess its safety and reflux control ability in comparison to standard microcatheters.

In our study, 85 % of the patients had nontarget embolization by beads reflux in the control group compared with only 20 % patients in the test group at M1. This nontarget embolization in the control group had enough beads to cause one case of ischemic cholangitis and one case of biloma, which remained without consequences but which could have been more serious [19].

Multifocal disease was more frequent in the test group (*p* = 0.05) which can be discussed as a factor of beads reflux seeing the need of multiple feeding artery selection. However, reflux was still less frequent in the test group. This hypothesis needs validation in a more homogenous population comparing multifocal disease only.

Reflux may have already been happening with DEB-TACE but it is only now being revealed by the use of radiopaque beads. Nontarget embolization was visualized at H0 and even more so on follow-up in the control group at M1, after elimination of the trapped iodine contrast. This may be explained by possible migration of the beads from the intra-arterial suspension at its proximal end into adjacent vessels. “Isolated” hepatic arteries [30,31] are known to be somehow involved in HCC recurrence after TACE [30] but might also explain the unusual beads distribution in some patients of our study. Intra or inter segmental/lobar arteries [7,10] are also known and described allowing reperfusion of a hepatic zone when the physiological flow is compromised, and can thus be involved here by retrograde flow. This is yet to be determined.

Beads distribution inside the HCC nodules changed over time in 39 % of the nodules (13/33) when graded according to Aliberti et al.’s classification. We argue that this is due to gradual physiologic elimination of trapped iodine contrast along with beads from the treated nodules. Trapped contrast material cannot be distinguished from the beads at H0. This puts into question Aliberti's grading system at H0 and could explain the mismatch at M1. We thereby recommend assessing NTE and using this grading system at M1 during the control CT after elimination of the trapped iodine.

Unlike traditional anti-reflux microcatheters, the SeQure is a “reflux control” microcatheter without any occluding device at its tip. The natural arterial flow is thus preserved, and the charged beads are driven to the target by the normal blood flow without any high-pressure injection resulting in opening of arterial shunts. The latter are a network of arterial intra or extrahepatic collaterals solicited in case of arterial occlusion [7,10,32,33].

This “reflux control” microcatheter, in comparison to traditional anti-reflux flow-directed microcatheters, would probably allow equivalent optimal delivery of beads and preventing reflux while preserving physiological blood flow. A comparative study between both devices is yet to be done.

The distribution of the beads follows thus the natural physiologic arterial pathways: their presence in adjacent segments is thereby due to reflux and not to the opening of arterial shunts.

The decrease in grading in beads distribution was not associated to treatment success or failure, as viable nodules were found among grade 1 nodules and nonviable tumors in grade 3 nodules, and the Fisher test's two-sided p-value was 0.15 (< 0.05), thus confirming that the two variables were independent. Comparison of LI-RADS score of grade 3 versus non-grade 3 nodules at M1 with another Fisher test has a p-value of 0.006 (<0.05) furthermore confirming the independence of those variables. This might be better evaluated or even predicted using artificial intelligence protocols adapted to HCC and liver treatment evaluation [34,35].

Microcatheter occlusion occurred in four cases with the SeQure microcatheter and loaded DC Bead LUMI, but in none with the standard microcatheters, however without any clinical repercussion. This can be explained by the small size of the beads (75 – 150 µm) allowing simultaneous passage of multiple beads side by side through the catheter lumen, combined with a decreased thrust through the end hole due to contrast exteriorization via the side holes. It does not seem to be related to the increased density of the beads following the chemical modification responsible for its radiopacity [14]. The 100 % iodine contrast dilution of the beads also leads to a highly viscose emulsion making it prone to aggregate formation [26]. This issue can be studied and the hypothesis validated in an in-vitro lab model along with the suggested hypothesis behind the beads migration between H0 and M1.

One obvious limit of our findings is the low number of patients in the cohort, also impacting univariate analyses, which did not reveal any significant risk factors. The single-center nature of the study is another limit. Nevertheless, the DEB-TACE procedures were performed by six different attending interventional radiologists. The atypical randomization process creates also a bias of inclusion even if both groups have no significant differences besides the MELD score and the unifocal/multifocal disease.

Further evaluation seems warranted.

## 5 Conclusion

This pilot study shows that beads reflux in DEB-TACE is real, significant and a potential source of complications. It is a proof of concept of this new reflux control microcatheter with encouraging results in hyperselective targeting and limiting beads reflux during embolization in DEB-TACE using radiopaque beads in HCC patients. Nevertheless, further validation with a larger multicenter study is required.

## CRediT authorship contribution statement

**Youssef Zaarour:** Data curation, Formal analysis, Investigation, Methodology, Writing – original draft, Writing – review & editing. **Haytham Derbel:** Formal analysis, Investigation, Methodology, Validation. **Charles Tran:** Data curation, Writing – original draft. **Laetitia Saccentia:** Investigation, Methodology. **Benjamin Longère:** Investigation, Methodology. **Maxime Blain:** Investigation, Methodology. **Giuliana Amaddeo:** Investigation, Methodology. **Alain Luciani:** Conceptualization, Methodology, Project administration, Validation, Writing – review & editing. **Hicham Kobeiter:** Conceptualization, Methodology, Project administration, Validation, Writing – review & editing. **Vania Tacher:** Conceptualization, Methodology, Project administration, Validation, Writing – original draft, Writing – review & editing.

## Declaration of competing interest

The authors declare that they have no known competing financial interests or personal relationships that could have appeared to influence the work reported in this paper.
